# Supplementary material for: PNPLA3-I148M Variant Promotes the Progression of Liver Fibrosis by Inducing Mitochondrial Dysfunction
Source: Int J Mol Sci. 2023 Jun 2;24(11):9681. doi: 10.3390/ijms24119681 (PMC10253263; doi:10.3390/ijms24119681)
Supplement: Supplementary file 1 [file ijms-24-09681-s001.zip › ijms-2405610-supplementary.pdf]

PNPLA3-I148M Variant Promotes the Progression of Liver Fibrosis by Inducing Mitochondrial Dysfunction

Yusong Gou [1,2,†], Lifei Wang [3,†], Jinhan Zhao [1,2], Xiaoyi Xu [1,2], Hangfei Xu [1,2], Fang Xie [2,4], Yanjun Wang [2,4], Yingmei Feng [5], Jing Zhang [1,*] and Yang Zhang [2,4,*]

1 The Third Unit, The Department of Hepatology, Beijing Youan Hospital, Capital Medical University, Beijing 100069, China; yusonggou@ccmu.edu.cn (Y.G.); zhaojinhan@mail.ccmu.edu.cn (J.Z.); xiaoyixu2020@163.com (X.X.); xuhangfei@mail.ccmu.edu.cn (H.X.)

2 Beijing Institute of Hepatology, Beijing Youan Hospital, Capital Medical University, Beijing 100069, China; xfxf815@126.com (F.X.); yjunwang@ccmu.edu.cn (Y.W.)

3 NHC Key Laboratory of Biotechnology of Antibiotics, Institute of Medicinal Biotechnology, Chinese Academy of Medical Sciences & Peking Union Medical College, Beijing 100050, China; lifeiwang2002@hotmail.com

4 Beijing Engineering Research Center for Precision Medicine and Transformation of Hepatitis and Liver Cancer, Beijing 100069, China

5 Beijing Youan Hospital, Capital Medical University, Beijing 100069, China; yingmeif13@sina.com

* Correspondence: zjyouan@ccmu.edu.cn (J.Z.); yang518@mail.ccmu.edu.cn (Y.Z.)

† These authors contributed equally to this work and shared first authorship.

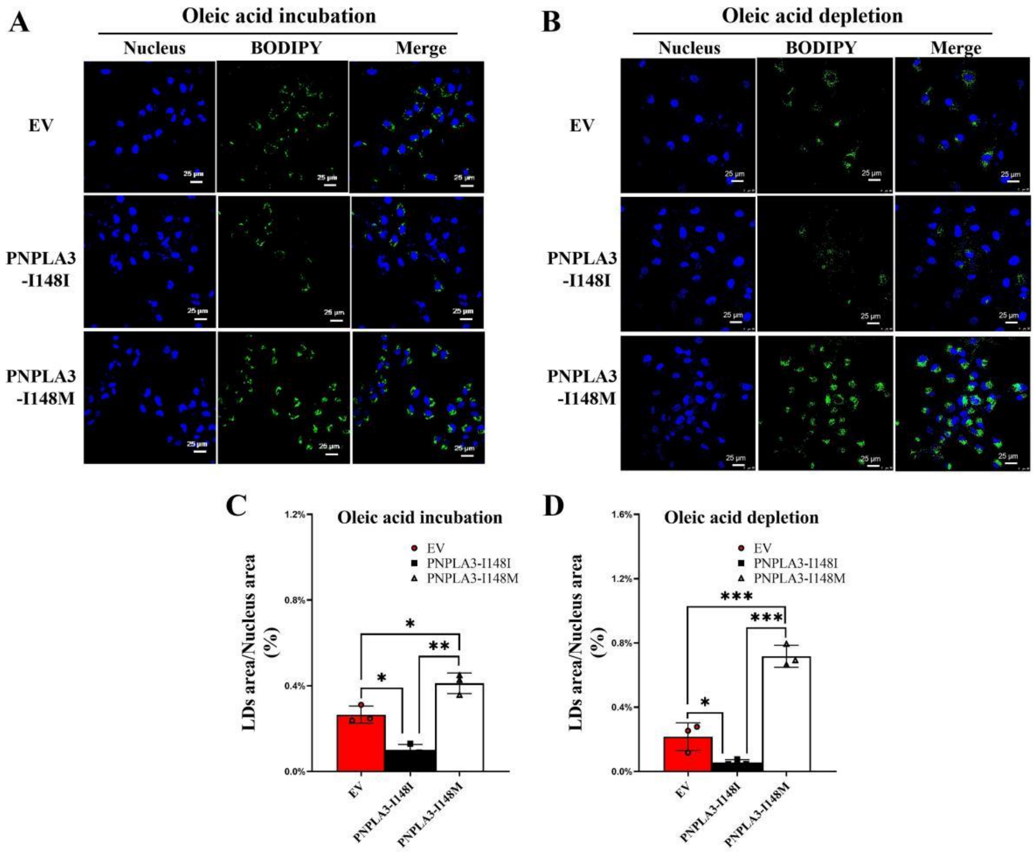

Supplementary Figure S1. Overexpression of PNPLA3-I148M promote lipids accumulation in LX-2 cells carrying EV, PNPLA3-I148I or I148M under OA-retinol incubation and depletion conditions. Distribution of lipid droplets in LX-2 cells stably overexpressing EV, PNPLA3-I148I and I148M under OA-retinol incubation (A) and depletion (B) conditions. blue: nucleus, green: lipid droplets. Scale bars: 25 μm, 400x magnification (C, D) Quantification of lipid droplets area in LX-2 cells.n=3, *P<0.05, **P<0.01, ***P<0.001. EV, empty vector; OA: Oleic acid.

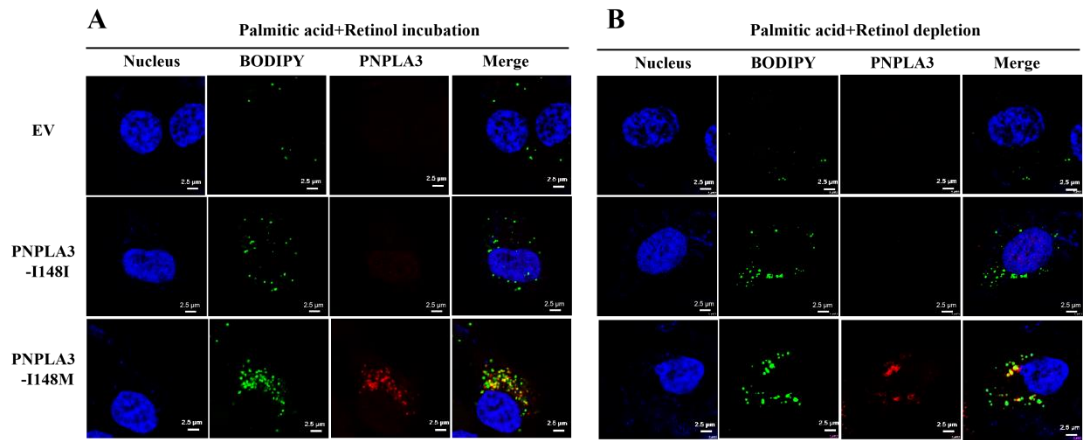

Supplementary Figure S2. Localization of PNPLA3 protein in LX-2 cells. (A, B) Localization of PNPLA3 in LX-2 cells under PA-retinol incubation and depletion conditions. blue: nucleus, green: lipid droplets, red: PNPLA3 protein. Scale bars: 2.5 μm, 1000x magnification. n=3. EV, empty vector; PA: Palmitic acid.

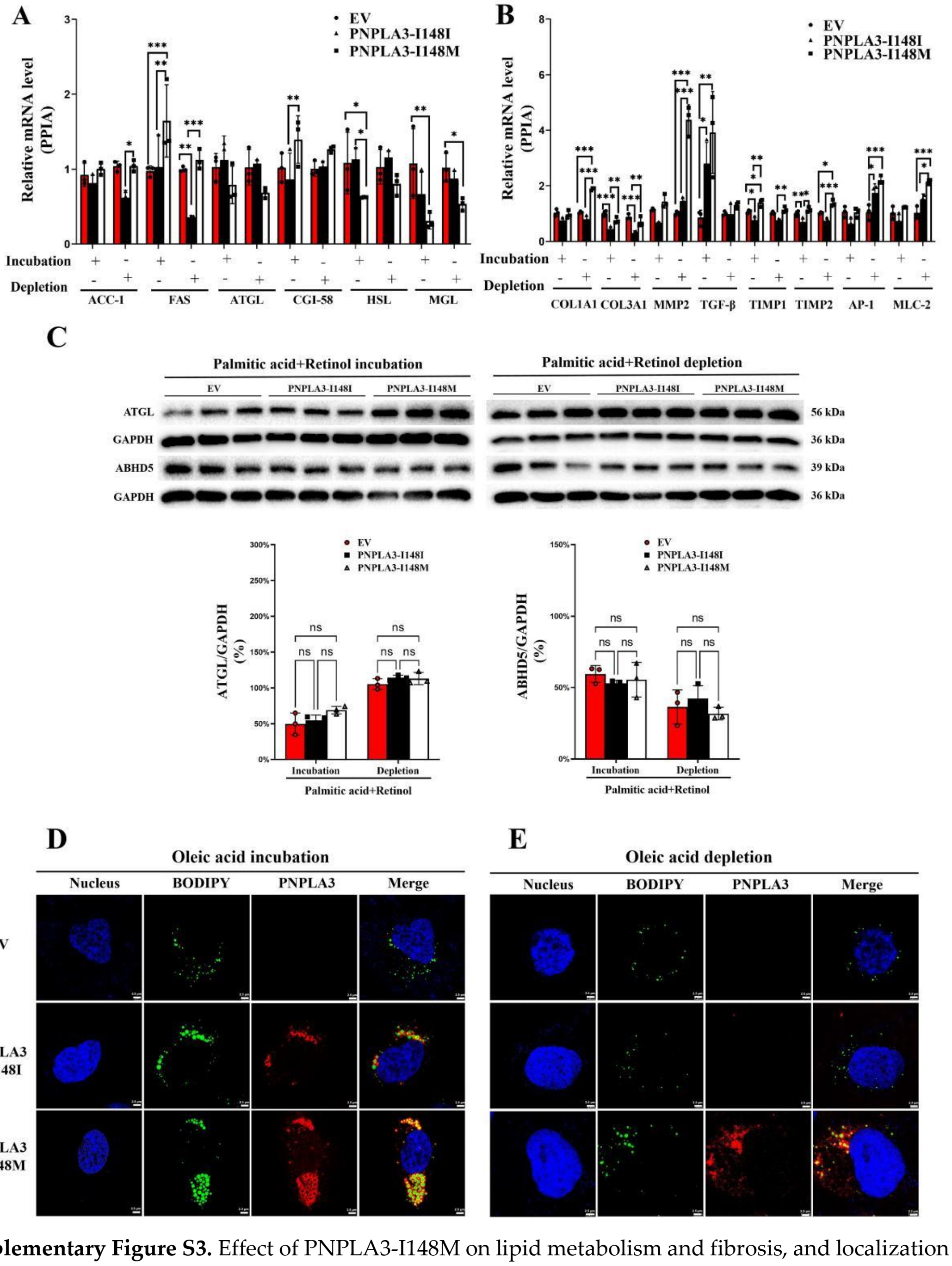

Supplementary Figure S3. Effect of PNPLA3-I148M on lipid metabolism and fibrosis, and localization of PNPLA3 protein in LX-2 cells. Real-time PCR analysis of lipid metabolism- (A) and fibrosis- (B) related genes in each group under PA-retinol incubation and depletion conditions. (C) Western blot analysis and quantification of ATGL and ABHD5 protein. (D-E) Localization of PNPLA3 in LX-2 cells under OA incubation and depletion conditions. blue: nucleus, green: lipid droplets, red: PNPLA3 protein. Scale bars: 2.5 μm, 1000 × magnification. n=3, *P<0.05, **P<0.01, ***P<0.001. EV, empty vector.

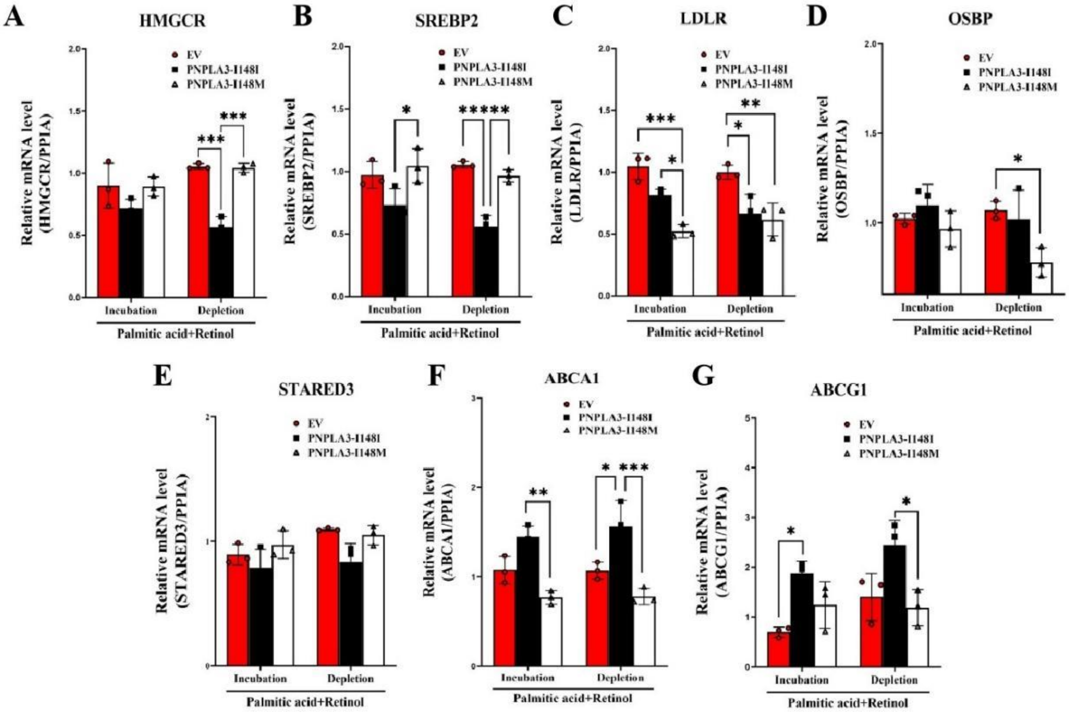

Supplementary Figure S4. Quantification of free cholesterol and mRNA expression of cholesterol metabolism-related genes *HMGCR* (**A**), *SREBP2* (**B**), *LDLR* (**C**), *OSBP* (**D**), *STARED3* (**E**), *ABCA1* (**F**) and *ABCG1* (**G**). n=3, *P<0.05, **P<0.01, ***P<0.001.

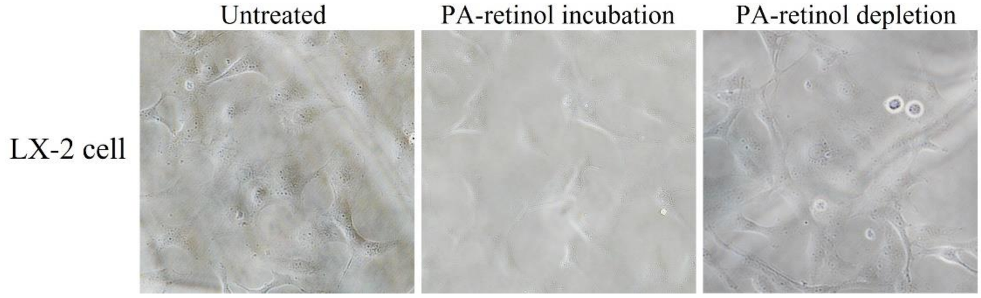

Supplementary Figure S5. Images of LX-2 under stimulation conditions with untreated, PA-retinol incubation and depletion.

Table S1. List of oligonucleotide primer

Gene	Forward primer (5’ -> 3’)	Reverse primer (5’ -> 3’)
ACC-1	TCGTTGTCATGGTCACACCT	CAGGCACTGGCACATAGTGA
FASN	AGATTGTGTGATGAAGGACATGG	TGTTGCTGGTGAGTGTGCATT
HSL	TCAGTGTCTAGGTCAGACTGG	AGGCTTCTGTTGGGTATTGGA
MGL	ATGCCAGAGGAAAGTTCCCC	CGTCTGCATTGACCAGGTG
ATGL	ATGGTGGCATTTCAGACAACC	CGGACAGATGTCACTCTCGC
ABHD	ACAGACCTGTCTATGCTTTTGAC	AGGGCACATCTCCACTCTTCA
COL1A1	GAGGGCCAAGACGAAGACATC	CAGATCACGTCATCGCACAAC
COL3A1	GGAGCTGGCTACTTCTCGC	GGGAACATCCTCCTTCAACAG
MMP2	GATACCCCTTTGACGGTAAGGA	CCTTCTCCCAAGGTCCATAGC
TIMP1	ACCACCTTATACCAGCGTTATGA	GGTGTAGACGAACCGGATGTC
TIMP2	AAGCGGTCAGTGAGAAGGAAG	GGGGCCGTGTAGATAAACTCTAT
TGF-β	GGCCAGATCCTGTCCAAGC	GTGGGTTTCCACCATTAGCAC
AP-1	AACAGGTGGCACAGCTTAAAC	CAACTGCTGCGTTAGCATGAG
MLC-2	TTGGGCGAGTGAACGTGAAAA	CCGAACGTAATCAGCCTTCAG
HMGCR	TGATTGACCTTTCCAGAGCAAG	CTAAAATTGCCATTCCACGAGC
SREBP2	AACGGTCATTCACCCAGGTC	GGCTGAAGAATAGGAGTTGCC
ABCA1	GGAGACGACAAATCTATGCAGTG	CCCAACAATGAGTTTCACGAGT
ABCG1	ATTCAGGGACCTTTCCTATTCGG	CTCACCACTATTGAACTTCCCG
OSBP	TCGTTGTCATGGTCACACCT	TCTCCAGGTGATTATGCTGCTT
LDLR	ACGGCGTCTCTTCCTATGACA	CCCTTGGTATCCGCAACAGA
STARD3	GGGCCATCTCTGATGTCCG	GCCTGTGTTGGTATTCAGTTCG
PINK1	CCCAAGCAACTAGCCCCTC	GGCAGCACATCAGGGTAGTC
BNIP3	CAGGGCTCCTGGGTAGAACT	CTACTCCGTCCAGACTCATGC
FIS1	GATGACATCCGTAAAGGCATCG	AGAAGACGTAATCCCGCTGTT
DRP1	CTGCCTCAAATCGTCGTAGTG	GAGGTCTCCGGGTGACAATTC
MFN1	TGGCTAAGAAGGCGATTACTGC	TCTCCGAGATAGCACCTCACC
MFN2	CACATGGAGCGTTGTACCAG	TTGAGCACCTCCTTAGCAGAC
SIRT1	TGTGTCATAGGTTAGGTGGTGA	AGCCAATTCTTTTTGTGTTCGTG
OPA1	CGACCCCAATTAAGGACATCC	GCGAGGCTGGTAGCCATATTT
AMPK	TTGAAACCTGAAAATGTCCTGCT	GGTGAGCCACAACTTGTTCTT
NRF1	GCTGATGAAGACTCGCCTTCT	TACATGAGGCCGTTTCCGTTT
NRF2	TCCAGTCAGAAACCAGTGGAT	GAATGTCTGCGCCAAAAGCTG
SOD1	GGTGGGCCAAAGGATGAAGAGT	CCACAAGCCAAACGACTTCC
TFAM	ATGGCGTTTCTCCGAAGCAT	TCCGCCCTATAAGCATCTTGA
UCP-2	CCCCGAAGCCTCTACAATGG	CTGAGCTTGGAATCGGACCTT
UCP-3	TGTTTTGCTGACCTCGTTACC	GACGGAGTCATAGAGGCCGAT
PPIA	CCCACCGTGTTCTTCGACATT	GGACCCGTATGCTTTAGGATGA
GAPDH	CTGGGCTACACTGAGCACC	AAGTGGTCGTTGAGGGCAATG

Table S2. List of antibodies

Antibodies	Source	Company	Catalog
ABCA1	Rabbit	ABclonal	A7228
ABCG1	Rabbit	ABclonal	A17907
ABHD5	Rabbit	ABclonal	A8673
ACAT	Rabbit	ABclonal	A13273
ATGL	Rabbit	ABclonal	A5126
DRP1	Rabbit	ABclonal	A21968
DYKDDDDK Tag (Flag)	Rabbit	CST	#2368
GAPDH	Rabbit	CST	#2118
LDLR	Rabbit	ABclonal	A20808
MFN2	Rabbit	ABclonal	A19678
PNPLA3	Rabbit	Abcam	ab81874
SCAP	Rabbit	Abcam	ab125186
SOD1	Rabbit	ABclonal	A12537
SREBP2	Rabbit	ABclonal	A13049
β -actin	Rabbit	CST	#4970
